# Active surveillance of oesophageal cancer after response to neoadjuvant chemoradiotherapy: dysphagia is uncommon

**DOI:** 10.1093/bjs/znad211

**Published:** 2023-07-07

**Authors:** Maria J Valkema, Manon C W Spaander, Jurjen J Boonstra, Jolanda M van Dieren, Wouter L Hazen, G Willemien Erkelens, I Lisanne Holster, Andries van der Linden, Klaas van der Linde, Liekele E Oostenbrug, Rutger Quispel, Erik J Schoon, Peter D Siersema, Michail Doukas, Ben M Eyck, Berend J van der Wilk, Pieter C van der Sluis, Bas P L Wijnhoven, Sjoerd M Lagarde, J Jan B van Lanschot

**Affiliations:** Department of Surgery, Erasmus MC Cancer Institute, Rotterdam, The Netherlands; Department of Gastroenterology and Hepatology, Erasmus MC Cancer Institute, Rotterdam, The Netherlands; Department of Gastroenterology and Hepatology, Leiden University Medical Centre, Leiden, The Netherlands; Department of Gastrointestinal Oncology, The Netherlands Cancer Institute, Amsterdam, The Netherlands; Department of Gastroenterology, Elisabeth Tweesteden Hospital, Tilburg, The Netherlands; Department of Gastroenterology, Gelre Hospital, Apeldoorn, The Netherlands; Department of Gastroenterology, Maasstad Hospital, Rotterdam, The Netherlands; Department of Gastroenterology, Zorggroep Twente, Almelo, The Netherlands; Department of Gastroenterology, Medical Centre Leeuwarden, Leeuwarden, The Netherlands; Department of Gastroenterology and Hepatology, Zuyderland Medical Centre, Heerlen, The Netherlands; Department of Gastroenterology and Hepatology, Reinier de Graaf Group, Delft, The Netherlands; Department of Gastroenterology and Hepatology, Catharina Hospital Eindhoven, Eindhoven, The Netherlands; Department of Gastroenterology and Hepatology, Radboud University Medical Centre, Radboud Institute for Health Sciences, Nijmegen, The Netherlands; Department of Pathology, Erasmus MC Cancer Institute, Rotterdam, The Netherlands; Department of Surgery, Erasmus MC Cancer Institute, Rotterdam, The Netherlands; Department of Surgery, Erasmus MC Cancer Institute, Rotterdam, The Netherlands; Department of Surgery, Erasmus MC Cancer Institute, Rotterdam, The Netherlands; Department of Surgery, Erasmus MC Cancer Institute, Rotterdam, The Netherlands; Department of Surgery, Erasmus MC Cancer Institute, Rotterdam, The Netherlands; Department of Surgery, Erasmus MC Cancer Institute, Rotterdam, The Netherlands

## Abstract

**Background:**

Active surveillance is being investigated as an alternative to standard surgery after neoadjuvant chemoradiotherapy for oesophageal cancer. It is unknown whether dysphagia persists or develops when the oesophagus is preserved after neoadjuvant chemoradiotherapy. The aim of this study was to assess the prevalence and severity of dysphagia during active surveillance in patients with an ongoing response.

**Methods:**

Patients who underwent active surveillance were identified from the Surgery As Needed for Oesophageal cancer (‘SANO’) trial. Patients without evidence of residual oesophageal cancer until at least 6 months after neoadjuvant chemoradiotherapy were included. Study endpoints were assessed at time points that patients were cancer-free and remained cancer-free for the next 4 months. Dysphagia scores were evaluated at 6, 9, 12, and 16 months after neoadjuvant chemoradiotherapy. Scores were based on the European Organisation for Research and Treatment of Cancer oesophago-gastric quality-of-life questionnaire 25 (EORTC QLQ-OG25) (range 0–100; no to severe dysphagia). The rate of patients with a (non-)traversable stenosis was determined based on all available endoscopy reports.

**Results:**

In total, 131 patients were included, of whom 93 (71.0 per cent) had adenocarcinoma, 93 (71.0 per cent) had a cT3–4a tumour, and 33 (25.2 per cent) had a tumour circumference of greater than 75 per cent at endoscopy; 60.8 to 71.0 per cent of patients completed questionnaires per time point after neoadjuvant chemoradiotherapy. At all time points after neoadjuvant chemoradiotherapy, median dysphagia scores were 0 (interquartile range 0–0). Two patients (1.5 per cent) underwent an intervention for a stenosis: one underwent successful endoscopic dilatation; and the other patient required temporary tube feeding. Notably, these patients did not participate in questionnaires.

**Conclusion:**

Dysphagia and clinically relevant stenosis are uncommon during active surveillance.

## Teaser

Dysphagia appears uncommon in patients with a clinically complete response until 16 months after neoadjuvant chemoradiotherapy according to the ChemoRadiotherapy for Oesophageal cancer followed by Surgery Study (‘CROSS’) regimen. In patients who might choose between standard surgery and active surveillance, the occurrence of persisting dysphagia during active surveillance is not an issue that needs to be taken into account.

## Introduction

One of the treatment options for patients with locally advanced resectable oesophageal cancer is neoadjuvant chemoradiotherapy (nCRT) followed by surgery^1^. Approximately one-third of patients have no residual tumour in the resection specimen after nCRT^2,3^. Therefore, in patients with a clinically complete response (cCR) after completion of nCRT, active surveillance might be an alternative to standard surgery^4,5^. This is currently being investigated in two randomized controlled trials^6–8^. During active surveillance, patients are offered surgery only when locoregional residual cancer is detected during clinical response evaluations (CREs) in the absence of distant metastases. In this way, a proportion of patients will avoid invasive surgery, with its associated risks of perioperative mortality, postoperative morbidity, and long-term decreased quality of life^9,10^.

It might well be that dysphagia persists or develops when the oesophagus is preserved after nCRT for locally advanced oesophageal cancer. It can be hypothesized that patients develop dysphagia due to chemoradiotherapy-induced stenosis and/or fibrotic dysmotility of the irradiated part of the oesophagus. The occurrence of dysphagia would be disadvantageous for patients who are cured by nCRT, but eventually might require repeated (semi-)invasive interventions for severe dysphagia or clinically relevant stenosis, such as dilatation, stent placement, or even surgery.

After standard oesophagectomy, some 40 per cent of patients, especially those with a cervical anastomosis, will be at risk of dysphagia caused by the development of benign and often refractory strictures^11–13^. Whether dysphagia occurs after nCRT, when the oesophagus is preserved, has not been evaluated previously. Such information should be shared with patients at the time of decision-making for active surveillance *versus* standard oesophagectomy. The topic of dysphagia relating to an organ-preserving strategy after nCRT should be evaluated in a specific subgroup, that is in patients without evidence of residual tumour in the oesophagus during active surveillance. This allows evaluation of dysphagia at time points without any interference of tumour regrowth. Therefore, we conducted a side study of the Dutch Surgery As Needed for Oesophageal cancer (SANO) trial. The first aim of the present study was to examine the prevalence and severity of dysphagia during active surveillance in patients with an ongoing cCR after nCRT. The second aim was to assess the rate of (non-)traversable stenosis after nCRT in these patients.

## Methods

### Study design

This was a retrospective multicentre cohort study using data from the Dutch prospective SANO trial^6^. Medical ethical approval for the present side study was obtained from the Erasmus MC (MEC-2022-0172). The requirement for additional informed consent was waived.

### Patients

Eligible patients with an adenocarcinoma or squamous cell carcinoma of the oesophagus or gastro-oesophageal junction, who underwent nCRT followed by active surveillance when they reached a cCR at 3 months after nCRT, were identified from the SANO trial database^6^. A cCR was defined as no evidence of residual tumour based on histopathological examination of biopsies (either regular or bite-on-bite biopsies^14^), endoscopic ultrasonography with fine-needle aspiration (EUS-FNA) of suspected lymph nodes, and no distant metastases on ^18^F-fluorodeoxyglucose (FDG) PET/CT. Patients were included in the present study when they had a cCR until at least 6 months after nCRT. This allowed the evaluation of dysphagia in patients who were cancer-free at the primary tumour location during at least one CRE after having started active surveillance.

### Neoadjuvant treatment

All patients who were included were scheduled for nCRT according to the ChemoRadiotherapy for Oesophageal cancer followed by Surgery Study (CROSS^2^) regimen. This regimen comprises five cycles of weekly carboplatin/paclitaxel and concurrent 41.1 Gy radiotherapy in 23 fractions.

### Active surveillance

Active surveillance was conducted according to the SANO trial protocol^6,7^. Briefly, CREs were performed repeatedly every 3 months in the first year and every 4 months in the second year, with intervals for CREs becoming longer up to 5 years after nCRT. The first CRE performed at 4–6 weeks after nCRT consisted of endoscopy with (bite-on-bite) biopsies and ^18^F-FDG PET/CT in case of proven residual tumour. All following CREs, starting at 3 months after nCRT, consisted of ^18^F-FDG PET/CT to exclude distant metastases, and endoscopy with random (bite-on-bite) biopsies of the primary tumour area and targeted biopsies of suspected lesions in the oesophagus. Endoscopy was combined with EUS-FNA to sample suspected lymph nodes.

### Severity of dysphagia

The course of any dysphagia during active surveillance was evaluated using the dysphagia domain of the European Organisation for Research and Treatment of Cancer oesophago-gastric quality-of-life questionnaire (EORTC QLQ-OG25). Questionnaires were sent to patients at predefined time points. The questionnaires at pretreatment and at 3, 6, 9, 12, and 16 months after nCRT were used. For the dysphagia domain, patients reported three items, focusing on problems with consuming solid food, semi-solid food, and liquids. Each of the items has four answer options on a 1–4 scale (1, ‘not at all’; 2, ‘a little’; 3, ‘quite a bit’; and 4, ‘very much’). An overall dysphagia score ranging between 0 and 100 was calculated according to the EORTC manual^15^. A score near 100 indicates severe symptoms; a score near 0 indicates no symptoms. Questionnaires were only used when patients did not have evidence of residual tumour in the oesophagus at that moment and not within the next 4 months, to exclude dysphagia that was caused by cancer regrowth. Thus, a minimum follow-up of 20 months was required for all patients to be able to analyse dysphagia scores until 16 months after nCRT.

### Clinically relevant stenosis

The number of patients who had clinically relevant stenosis during active surveillance was determined using the endoscopy reports. Clinically relevant stenosis was defined as a stenosis for which therapeutic intervention was performed at least once. For patients with clinically relevant stenosis, symptoms, severity of stenosis, type of intervention, number of required interventions, and complications were described.

### Stenosis after neoadjuvant chemoradiotherapy

The presence of (non-)traversable stenoses at the primary tumour location was analysed for all patients, for all CREs, that is at 3, 6, 9, 12, and 16 months after nCRT. For this purpose, the endoscopy reports were used. Any description of ‘stenosis’, ‘narrowing’, ‘stricture’, ‘fibrotic ring’, and ‘tapering of the lumen’ was noted as ‘stenosis’. This was labelled as ‘stenosis, easily traversable’, ‘stenosis, traversable with pressure’, or ‘non-traversable stenosis’, depending on the description in the report. When the endoscopy report did not mention any of the previous criteria and the endoscopic evaluation included the stomach, the assessment was not labelled as stenosis. ‘Subtle scarring’, ‘irregular mucosa’, and ‘(slight) oedematous mucosa’, without any obvious description of stenosis or narrowing, were noted as ‘no stenosis’. CREs at which there was cancer regrowth in the oesophagus at that time point or within the next 4 months were excluded from analysis. Also, only CREs were considered at which there had not yet been an intercurrent intervention for a clinically relevant stenosis.

### Statistical analysis

Frequencies and percentages were used to describe the prevalence of clinically relevant stenoses, therapeutic interventions, and (non-)traversable stenoses. Baseline clinical and tumour characteristics were compared between patients with and without a (non-)traversable stenosis, using Fisher’s exact test for categorical variables and a Mann–Whitney *U* test for continuous variables.

Dysphagia scores were reported for each CRE as the median (interquartile range (i.q.r.)) and mean(s.d.). Patients with outlying scores were described in the context of available follow-up questionnaires and self-reported symptoms at hospital visits. Outlying scores were defined as median scores greater than 33.3, meaning that a little dysphagia was reported for all domains (that is solid food, semi-solid food, and liquids) or that quite a bit or very much dysphagia was reported for at least one of the domains.

Statistical analysis was performed using R version 4.2.2 (R: A Language and Environment for Statistical Computing; The R Foundation for Statistical Computing, Vienna, Austria). The code can be accessed via github.com/mjvalkema/dysphagia.

## Results

### Patients

In total, 131 patients with a persistent cCR for at least 6 months after nCRT were included in the study. Baseline characteristics are shown in *Table 1*.

**Table 1:** Baseline characteristics of patients included in the study; *n* = 131

Baseline characteristic	Value
Age (years), median (i.q.r.)	69.1 (63.7–73.1)
Sex, male	100 (76.3)
**Histology**	
Adenocarcinoma	93 (71.0)
Squamous cell carcinoma	36 (27.5)
Other	2 (1.5)
**Tumour location**	
Proximal oesophagus	2 (1.5)
Middle oesophagus	18 (13.7)
Distal–GOJ	111 (84.7)
**Tumour differentiation**	
Good–moderate	87 (66.4)
Poor	39 (29.8)
Unknown	5 (3.8)
**cT stage**	
cT2	27 (20.6)
cT3	91 (69.5)
cT4a	2 (1.5)
cTx	11 (8.4)
**cN stage**	
cN0	60 (45.8)
cN1	46 (35.1)
cN2	15 (11.5)
cN3	3 (2.3)
cNx	13 (5.3)
**Tumour circumference (endoscopy)**	
0–25%	15 (11.5)
26–50%	33 (25.2)
51–75%	22 (16.8)
>75%	33 (25.2)
Unknown	28 (21.3)
**Tumour length (cm on endoscopy), median (i.q.r.)**	5.0 (3.0–6.0)
**Tumour length (cm on CT), median (i.q.r.)**	4.7 (4.0–6.0)

Values are *n* (%) unless otherwise indicated. Numbers may not add up to 100 per cent due to rounding. i.q.r., interquartile range; GOJ, gastro-oesophageal junction; cT stage, clinical tumour stage; cN stage, clinical nodal stage.

### Severity of dysphagia

Of the 131 patients who received QLQs, 114 (87.0 per cent) participated at least once. Questionnaires after nCRT were completed between April 2018 and June 2022. Response rates after nCRT varied between 60.8 and 71.0 per cent per CRE (*Table 2*). The pretreatment median dysphagia score was 11.1 (i.q.r. 11.1–33.3). Median dysphagia scores after nCRT did not exceed 0 at all subsequent time points until 16 months after nCRT (*Table 2*).

**Table 2:** Dysphagia scores based on the European Organisation for Research and Treatment of Cancer oesophago-gastric quality-of-life questionnaire (EORTC QLQ-OG25) for clinically complete responders after neoadjuvant chemoradiotherapy who underwent active surveillance

	Pretreatment	3 months	6 months	9 months	12 months	16 months
Median (i.q.r.)	11.1 (11.1–33.3)	0.0 (0.0–0.0)	0.0 (0.0–0.0)	0.0 (0.0–0.0)	0.0 (0.0–0.0)	0.0 (0.0–0.0)
Mean(s.d.)	21.7(19.0)	3.6(8.5)	1.3(4.0)	1.3(3.6)	1.9(4.8)	3.8(11.9)
Rate of participation*	65/131 (49.6%)	93/131 (71.0%)	77/118 (65.3%)	69/107 (64.5%)	66/104 (63.5%)	62/102 (60.8%)

The rate of participation is defined as the number of patients participating in the questionnaire at that particular time point divided by the total number of patients who did not develop a local recurrence in the oesophagus at that time point and not within the next 4 months. At pretreatment, only questionnaires were included that had been completed until a maximum of 7 days after the start of neoadjuvant chemoradiotherapy. i.q.r., interquartile range.

Few dysphagia scores greater than 33.3 were observed after nCRT. At 6 months after nCRT, this concerned one patient with a score of 44.4. Dysphagia resolved spontaneously 1 month later and was not reported again up to the last available questionnaire 4 months later. At 12 months after nCRT, another patient had a score of 66.7, which decreased to 22.2 at the last available questionnaire 5 months later without interventions. At 16 months after nCRT, two patients had a score of 44.4. One of these patients reported an increase in dysphagia 6 months later, but this was attributed to a combination of kyphoscoliosis, pectus excavatum, and a vertebral collapse as seen using diagnostic CT. In the other patient the score decreased to 22.2 at the last available questionnaire 7 months later.

### Clinically relevant stenosis

Of 131 patients, 2 (1.5 per cent) had a clinically relevant stenosis at least once during active surveillance. One patient had a persistent non-traversable stenosis from 9 until 24 months after nCRT (*Table 3*). This patient had dysphagia for solid food from 3 months onwards. It was decided not to perform dilatation. However, between 16 and 20 months after nCRT, the patient required temporary tube feeding because of weight loss due to an increase in dysphagia. Afterwards, until the last available follow-up at 24 months after nCRT, the patient reported dysphagia only for solid food without any other intervention having taken place. Another patient underwent dilatation with Savary bougies for a clinically relevant stenosis. This patient had a stenosis (traversable with pressure) 7 months after nCRT for which one dilatation was performed. After 1 year the patient again reported symptoms of dysphagia, but refused any further diagnostic examinations or dilatations. Dysphagia scores were unavailable for these two patients; both did not respond to questionnaires.

**Table 3:** Rates of stenosis per clinical response evaluation until 16 months after nCRT in patients without tumour recurrence during active surveillance

	3 months	6 months	9 months	12 months	16 months

**Stenosis, easily traversable**	15 (11.5)	6 (5.4)	8 (7.8)	7 (7)	7 (7)
dysphagia score, median (i.q.r.); mean(s.d.)	0 (0–11.1); 6.8(9.7)	5.6 (2.8–8.3); 5.6(7.9)	0 (0–2.8); 2.8(5.6)	5.6 (0–11.1); 5.6(6.4)	0 (0–0); 0(0)
Stenosis, traversable with pressure*	1† (1)	3†‡ (3)	0 (0)	0 (0)	0 (0)
Non-traversable stenosis*	0 (0)	0 (0)	1† (1)	1† (1)	1† (1)
**Total number of patients§**	131	111	102	98	97

Values are *n* (%) unless otherwise indicated. *No dysphagia scores were available for these patients as they did not participate in questionnaires. †Involves one patient with persistent dysphagia for solid food, but not for semi-solid food and liquids. Dilatation was not performed. ‡Involves one patient who underwent dilatation afterwards. Stenoses that were observed in further follow-up are censored from the table. §Defined as the number of patients with an available endoscopy report corresponding to the particular time point, without development of localized residual tumour within 4 months. i.q.r., interquartile range.

### Stenosis after nCRT

In total, 27 patients had a stenosis described in the endoscopy report at the primary tumour area at least once during active surveillance. This involved 26 of 131 (19.8 per cent) patients with a traversable stenosis and 1 patient with a non-traversable stenosis (see the previous paragraph for a description of this patient). A (non-)traversable stenosis was described in approximately 10 per cent of patients per CRE (*Table 3*). Clinical and tumour characteristics of the 27 patients with a stenosis were not significantly different from those of the 104 patients without any stenosis (*Table 4*).

**Table 4:** Patient and tumour characteristics for patients who had a stenosis at least once *versus* patients who did not

	No stenosis (*n* = 104)	Stenosis (*n* = 27)	P*
**Sex, male**	80 (76.9)	20 (74.1)	0.801
**Histology**			0.807
Adenocarcinoma	75 (72.1)	18 (66.7)	
Squamous cell carcinoma	28 (26.9)	8 (29.6)	
Other	1 (1.0)	1 (3.7)	
**Tumour location, proximal–mid**	16 (15.4)	4 (14.8)	1.000
**Tumour differentiation grade**			0.226
Good–moderate	73 (70.2)	14 (51.9)	
Poor	29 (27.9)	10 (37.0)	
Unknown	2 (1.9)	3 (11.1)	
**cT stage**			0.781
cT2	21 (20.2)	6 (22.2)	
cT3–4a	76 (73.1)	17 (63.0)	
cTx	7 (6.7)	4 (14.8)	
**Tumour circumference (endoscopy)**			0.223
>50%	41 (39.4)	14 (51.9)	
0–50%	41 (39.4)	7 (25.9)	
Unknown	22 (21.2)	6 (22.2)	
**Tumour length (cm on endoscopy), median (i.q.r.)**	4.5 (3.0–6.0)	5.0 (3.5–7.0)	0.178

Values are *n* (%) unless otherwise indicated. *Fisher’s exact test was performed between the known categories, that is without the ‘other’, ‘unknown’, and ‘cTx’ categories. cT stage, clinical tumour stage; i.q.r., interquartile range.

## Discussion

The present study demonstrates that dysphagia is uncommon in patients with an ongoing cCR until at least 16 months after nCRT. Dysphagia scores in these patients were close to 0 (that is no dysphagia) during active surveillance. Only two patients had clinically relevant stenosis during active surveillance and their symptoms were clinically manageable. Of note, both patients did not participate in questionnaires.

In previous studies, only short-term results were available up to 12 weeks after nCRT. It was shown that nCRT relieves dysphagia symptoms before standard oesophagectomy^16^. It was also shown that neoadjuvant chemotherapy (nCT) achieves effective reduction of dysphagia 1 month after completion of nCT^17^. However, patients in both studies did not undergo active surveillance. Long-term dysphagia outcomes before postponed surgery are not available so far.

In the one patient who was described with a clinically relevant stenosis and underwent endoscopic dilatation, treatment was successful after one intervention. By contrast, oesophageal stricture formation following standard oesophagectomy after nCRT, especially at the level of a cervical anastomosis, is a problem in up to 50 per cent of patients. Such strictures may cause severe dysphagia and can be refractory to dilatation^11–13^. In the long-term, however, self-reported dysphagia scores in patients without disease recurrence after surgery are generally low. At 4 years after oesophagectomy, with or without (neo)adjuvant therapy, mean EORTC QLQ-OG25 dysphagia scores range between 8 and 15^18^. For comparison, at 16 months after nCRT in the present study, the mean dysphagia score was 3.7, indicating the almost complete absence of dysphagia.

An important strength of this study is that patients were identified from a prospective trial, which ensures that CREs were systematically performed and monitored for adherence to the trial protocol^6^. A large proportion of patients also participated in a study using QLQs, allowing for a considerable sample size to describe the course of dysphagia scores over time. Furthermore, for all patients, all available endoscopy reports after nCRT were revised. As such, additional information on therapeutic interventions for dysphagia and the presence of stenoses could be reliably collected at the time points corresponding to the questionnaires, but also for unplanned follow-up visits in-between.

A limitation of the study is the questionnaire participation rate after nCRT, which ranged between 60 and 70 per cent. Consequently, self-reported dysphagia might have been underestimated. In addition, dysphagia outcomes in this study were limited to a maximum of 16 months after nCRT. Nevertheless, this interval probably contained the most relevant information regarding the effects of radiotherapy-induced inflammation on dysphagia (that is dysphagia caused by swelling), as this inflammation is expected to resolve within the first year of active surveillance in patients with an ongoing cCR^19^. Furthermore, recent data have shown that 45 per cent of patients with stenosis early after nCRT and standard oesophagectomy have a complete response in the resection specimen^20^. For the subgroup of patients with such stenosis, it remains unknown whether the stenosis, with or without dysphagia, would have persisted if they had undergone active surveillance (with or without endoscopic dilatation) and remained cancer-free. However, according to the SANO trial protocol, the occurrence of a non-traversable stenosis will remain an indication for surgery, as full evaluation of the oesophagus and gastro-oesophageal junction should be possible during active surveillance^6^.

## Data Availability

The data sets generated and/or analysed during the present study are not publicly available, but are available from the corresponding author on reasonable request.
